# Patient-centred standards of care for adults with myositis

**DOI:** 10.1186/s41927-017-0002-7

**Published:** 2017-11-28

**Authors:** James B. Lilleker, Patrick Gordon, Janine A. Lamb, Heidi Lempp, Robert G. Cooper, Mark E. Roberts, Paula Jordan, Hector Chinoy, On behalf of the UK Myositis Network (UKMYONET), Robert G. Cooper, Christopher Edwards, David Hilton-Jones, Patrick Kiely, Heidi Lempp, Neil McHugh, Pedro Machado, John Pauling, Sarah Tansley, John Winer, Myositis UK

**Affiliations:** 1 Greater Manchester Neuroscience Centre, Salford Royal NHS Foundation Trust, Manchester Academic Health Science Centre, Stott Lane, Salford, UK; 2 NIHR Manchester Biomedical Research Centre, Central Manchester University Hospitals NHS Foundation Trust, The University of Manchester, Manchester, UK; 3 King’s College Hospital NHS Foundation Trust, London, UK; 4 Centre for Integrated Genomic Medical Research, School of Health Sciences, Faculty of Biology Medicine and Health, The University of Manchester, Manchester, UK; 5 Academic Rheumatology, Faculty of Life Sciences & Medicine, King’s College London, London, UK; 6 MRC-ARUK Institute for Ageing and Chronic Disease, University of Liverpool, Liverpool, UK; 7 Myositis UK, Southampton, UK; 8 Rheumatology Department, Salford Royal NHS Foundation Trust, Manchester Academic Health Science Centre, Stott Lane, Salford, UK

**Keywords:** Myositis, Idiopathic inflammatory myopathy, Delphi process, Standards of care, Patient-centred care, Quality improvement

## Abstract

**Background:**

The idiopathic inflammatory myopathies (IIM, myositis) are a heterogeneous group of chronic autoimmune disorders causing considerable physical and mental health impact. There is a lack of formalised guidance defining best practice for the management of myositis, contributing to inconsistent care provision and some patients feeling isolated and unsupported.

To address these issues, we evaluated the clinical services available to adults with myositis in the UK. We then created patient-centred standards of care using a structured process involving patients, their relatives and caregivers, physicians and allied healthcare professionals.

**Methods:**

After an initial focus group, the clinical services available to patients with myositis were evaluated using a patient-completed questionnaire. Draft standards of care were created, each addressing deficits in care provision identified by patients. In response to feedback, including a two-stage modified Delphi exercise, these draft standards were iteratively improved until consensus was reached. Accompanying plain language versions of the standards of care and an audit tool were also created.

**Results:**

We identified issues regarding diagnostic pathways, access to specialist services, advice and support regarding employment, medication-related adverse events and the treatment of extra-muscular manifestations. Fifteen standards of care were drafted. After modification, agreement was reached on eleven final standards of care.

**Conclusion:**

These patient-centred standards of care for adults with myositis provide a benchmark for the evaluation of local practice. Their implementation will promote consistent good practice across care providers and empower patients when seeking access to local services.

**Electronic supplementary material:**

The online version of this article (10.1186/s41927-017-0002-7) contains supplementary material, which is available to authorized users.

## Background

The idiopathic inflammatory myopathies (IIM, myositis) are a heterogeneous group of long-term autoimmune inflammatory conditions. Myositis negatively impacts the quality of life of an estimated 250,000 patients worldwide [1]. A lack of formalised guidance defining best practice contributes to inconsistent care provision and may influence clinical outcomes.

In circumstances where evidence-based or data-driven approaches are not possible, or not yet available, the production of standards of care allows healthcare professionals to benchmark their service using a set of agreed ‘minimum’ or ‘optimum’ consensus standards. This contrasts with the definition of a guideline, which is generated through a systematic evaluation of the available evidence and usually assists with clinical decision making in a specific scenario [2]. The Arthritis and Musculoskeletal Alliance (ARMA) have defined standards of care for patients with connective tissue diseases, but nothing similar exists to address the specific needs of adults with myositis [3].

Polymyositis (PM), and other myositis subtypes, are classified as orphan diseases (ORPHA:98,482). The rarity and heterogeneity of myositis means that delayed diagnosis and misdiagnosis occur commonly [4]. Most clinicians are unfamiliar with the management of these chronic conditions and patients can feel isolated and unsupported [5]. To overcome some of these problems we surveyed the experiences of adults in the UK living with myositis and using a structured process have created patient-centred standards of care.

## Methods

Six steps were undertaken to produce the final standards of care. These are summarised in Table 1.

**Table 1:** Steps taken to produce the patient-centred standards of care for adults with myositis

Step 1 - Initial information gathering from patients, relatives and caregivers
• Focus group at Myositis UK AGM, July 2014 (30 participants) • Patient completed service evaluation questionnaire, March 2015 ○ 151 responses obtained
Step 2 - Statement drafting
• 15 draft statements reflecting *optimum* standards of care created by steering team. • Each statement addressed perceived deficits in current care arrangements as identified by patients
Step 3 - Initial feedback
• Feedback on draft statements sought from: ○ Myositis UK AGM, July 2015 (75 participants) ○ Members of the UK Myositis Network (UKMYONET) (responses from 12 individuals received) • Statements updated. One statement removed from process
Step 4 - Modified Delphi exercise - Round one
• 14 updated draft statements presented electronically to the Delphi panel (December 2015). Responses from 25 individuals received. • Level of agreement and suggestions for improvement analysed: ○ Eight statements met predetermined consensus level (mean level of agreement ≥8.5) ○ Six statements failed to meet predetermined consensus level. These were updated based on 40 individual items of feedback
Step 5 - Modified Delphi exercise - Round two
• Updated statements (*n* = 6) re-presented to panel (April 2016) • Three statements reached predetermined consensus level. • Remaining three statements were removed from the process
Step 6 - Final production of the patient-centred Standard of Care
• Approved updated statements arranged into three themes (December 2016) • Minor changes to grammar and readability of three statements • Creation of suggested audit standards to accompany each statement • Creation of plain language versions of each statement (January 2017)

AGM = annual general meeting

### Initial information gathering from patients, relatives and caregivers

An initial focus-group of 30 participants (approximately 50% patients, 50% relatives/caregivers) was held at the Myositis UK (www.myositis.org.uk) Annual General Meeting (AGM) in July 2014. Information regarding the physical implications of disease and the social and emotional difficulties faced was gathered. These findings contributed to the development of a service evaluation questionnaire which was distributed by email and posted to all adult patient members of Myositis UK (*n* = 485) (Additional file 1 – Section One).

### Statement drafting and initial feedback

Responses to the service evaluation questionnaire were analysed and 15 draft statements produced by agreement of the steering team (JBL, HC, JAL). Each reflected an *optimum* expected standard of care to address a matter highlighted by patients in the service evaluation questionnaire. These draft statements were then presented to attendees at the Myositis UK AGM in July 2015 and to members of the UK Myositis Network (UKMYONET) by email. Updating the statements in response to oral and written feedback received was overseen and agreed amongst the steering team.

### Modified Delphi exercise

A modified, two-round Delphi consensus building exercise using a website-based survey system was then performed according to a pre-specified protocol. Briefly, the statements were sent to a multidisciplinary Delphi panel who were asked to indicate their level of agreement with each one using a ten point Likert scale, and to provide suggestions for improvement. A predetermined consensus level was agreed by the steering team (a mean agreement score ≥ 8.5 out of 10, unknown to the Delphi panel). Statements reaching this consensus level passed through to the final stage of the process. Where this level was not reached, suggestions for improvement were examined and incorporated into an updated version of the statements, which were then sent back to the panel. Those not reaching consensus at this point were removed from the list of standards. Twenty-eight patient representatives and healthcare professionals (including Allied Health Professionals, Rheumatologists and Neurologists) were invited to take part on the panel.

### Final production

The final standards of care were grouped into domains. Suggested audit standards were produced, and plain language versions of the standards in a checklist format were agreed after a teleconference focus-group with patients, caregivers and Myositis UK charity representatives.

### Statistics

A descriptive statistical analysis of the service evaluation questionnaire was performed (JBL). The mean and standard deviation (SD) were calculated where data were normally distributed, whereas medians and interquartile ranges (IQR) were calculated for non-normally distributed data. For hypothesis testing Fisher’s exact test or Mann Whitney rank-sum test were used where applicable. A *p*-value of <0.05 was considered as significant.

## Results

### Initial information gathering from patients, relatives and caregivers

In total, 151 responses (31% [151/485] return rate, 145 online and 6 by post) to the service evaluation questionnaire were obtained. Patients with dermatomyositis (DM) (37%), PM (27%) and inclusion body myositis (36%) participated. The mean age of respondents was 59 years (SD 14) and 33% were male. Diagnostic delays were common, particularly between presentation to the general practitioner and onwards referral to secondary care (median interval 2 months [IQR 0–4]). The majority of patients (58%) had been given at least one incorrect diagnosis prior to their final accurate diagnosis.

Satisfaction levels regarding access to Allied Healthcare Professionals were low to moderate (19% satisfied with access to rehabilitation services, 51% to physiotherapy and 55% to occupational therapy). Most respondents (83%) were unaware of any local support groups for patients with myositis. In addition, low levels of satisfaction were reported regarding the management of extra-muscular and non-medical aspects of the disease. For example, only 23% of patients were satisfied that they had received sufficient support relating to employment issues, and only 24% stated that psychological aspects had being adequately addressed.

Overall, 54% of patients reported that they had received satisfactory counselling about potential adverse effects of treatments and 64% were confident that they could obtain urgent medical advice regarding their myositis, e.g. in the event of a disease flare. Furthermore, 69% of those with IBM were reviewed by a specialist less than once annually. Only 33% of patients indicated that they had been invited to participate in myositis research studies.

### Statement drafting, feedback and updating

Fifteen draft statements were created, of which 13 were changed after feedback from attendees at the Myositis UK AGM 2015 (*n* = 75) and UKMYONET (12 responses, 57 individual items of feedback). One statement was removed from the process at this stage due to overwhelmingly negative feedback from both sources (Additional file 1 – Section Two).

Full results of the modified Delphi exercise are detailed in the Additional file 1 (Section Three). In the first round, responses were received from 24 of the 28 (86%) invited panel members. Eight of the 14 statements met the pre-determined consensus agreement level (a mean agreement score ≥ 8.5 out of 10). Forty items of feedback were received regarding the statements not reaching this consensus level. Analysis of this feedback informed statement updates, agreed amongst the steering team. During this process, one deviation from the protocol occurred where a statement was slightly refocussed by the steering team, to ensure that a key area of concern from the service evaluation questionnaire (the low levels of satisfaction regarding management of psychological aspects) was addressed.

In the second round of the Delphi exercise, 21 responses from 28 invitations (75%) were obtained. Despite the modifications, three statements failed to reach the pre-determined consensus level (mean agreement levels: 6.9, 8.1, 8.2) and were therefore removed from the process*.* The three remaining ones (mean agreement levels: 8.7, 9.1, 9.3) passed to the final stage.

### Final production

The final eleven statements underwent minor modifications to improve readability and accessibility (Table 2). A log of all such changes to the statements is included in the Additional file 1 (Section Four). These were then arranged in to three emerging themes: (i) presentation, referral and diagnosis; (ii) care arrangements and the interaction between myositis specialists and other healthcare professionals; (iii) disease management and holistic care. An audit tool with suggested audit standards (Additional file 1 - Section Five) and plain language versions of the statements were also created (Table 2).

**Table 2:** Final patient-centred standards of care for adults with myositis and accompanying plain language versions of the standards

Patient-centred standards of care			Plain language version of standards
*Domain 1: Presentation, referral and diagnosis*			
	Myositis should be considered in patients with *unexplained weakness, fatigue, rash, myalgia or arthralgia* • Testing of serum *CK is a useful screening tool*, but can be normal in some scenarios	*9.1*	When I first developed symptoms of myositis, was the correct diagnosis considered early?
	GPs should identify patients presenting with features of myositis (e.g. muscle weakness, raised CK +/− rash) and refer to a specialist *as soon as this diagnosis is considered*	*8.8*	Was I referred to an appropriate specialist quickly?
*Domain 2: Care arrangements and the interaction between myositis specialists and other healthcare professionals*			
	Patients with myositis should be *under the care of a specialist with specific expertise and experience in managing myositis* • This could be either directly or as part of a formal shared-care agreement with a local physician	*9.5*	Am I under the care of a specialist (either directly or under shared-care) who is experienced and competent in the management of patients with myositis?
	Patients with myositis should continue to be *periodically reviewed by a myositis specialist* for as long their disease is active or muscle strength continues to deteriorate • This could be either directly or as part of a formal shared-care agreement with a local physician	*9.1*	Do I see my specialist with sufficient frequency to meet my needs?
	The services for patients with myositis should include access to *ongoing specialist* physiotherapy, occupational therapy and speech and language therapy • This could be integrated in to the specialist clinic or via a formal shared-care agreement between specialist and non-specialist (local) therapists	*9.2*	Do I have adequate support from other health professionals (including physiotherapists, occupational therapists and speech and language therapists) that have experience in managing patients with myositis?
	There should be *clear protocols defining how* patients with myositis should *seek urgent advice.* • For example, the specialist centre might provide a *dedicated telephone advice line* for patients and other healthcare professionals	*8.9*	Should the need arise, am I able to obtain appropriate urgent medical advice regarding my myositis or its treatment?
*Domain 3: Disease management and holistic care*			
	When considering starting patients with myositis on immunosuppression*, detailed discussion regarding the potential benefits and possible side effects must take place*. • This could be reinforced by other members of the multidisciplinary team (e.g. pharmacist) • Formal shared-care agreements with GPs should also be in place	*8.8*	Before I am offered treatments for my myositis, do I understand the relevant benefits and potential risks?
	Healthcare professionals should specifically address *extra-muscular symptoms* such as pain, fatigue and depression at each consultation	*8.7*	Are issues such as pain, fatigue or low mood addressed in addition to my muscle weakness during consultations with my specialist?
	Services for patients with myositis should provide *holistic care* that addresses *physical and psychological aspects* of disease and its *social implications* • For example, this may include difficulties with employment	*8.7*	If required, do I have access to support with how myositis affects my day-to-day life (for example, the effect on my job)?
	Patients with myositis should be *signposted to appropriate information resources and patient groups*	*9.3*	Have I been made aware of relevant myositis information resources and patient groups?
	Patients with myositis should be *offered participation in clinical trials* as part of routine practice	*8.8*	Have I been offered participation in clinical trials for myositis?

Mean level of agreement is shown adjacent to each standard of care and is derived from responses on a ten point Likert scale. GPs = general practitioners

## Discussion

Our patient-centred approach has helped to create a set of unique standards of care for adults with myositis. By specifically addressing deficiencies in care highlighted by patients and then using an iterative process that considered feedback from patients, relatives, caregivers and healthcare professionals, we have produced standards of care tailored to the individual healthcare needs of patients with myositis.

The service evaluation questionnaire involved a wide variety of patients with myositis living in England, Scotland and Wales, thus capturing a variety of experiences and views. In addition to patient representatives, the Delphi panel also represented a range of healthcare professionals from diverse geographic locations, minimising bias from one professional group or location. However, there is the possibility that not all relevant stakeholders have been included and we have not included feedback or approval from those with juvenile-onset myositis (i.e. Juvenile-onset DM). It is acknowledged that some aspects of the produced standards may apply more to certain subgroups of myositis patient than others. Similarly, our work is derived from views regarding the UK healthcare system, and may therefore not be applicable or relevant in other locales.

Implementation of the standards would require additional resources, e.g. to improve access to Allied Healthcare Professionals, such as physiotherapists who have experience in caring for and managing patients with myositis. However, other standards could be quickly implemented, e.g. an up-to-date list of ongoing myositis clinical trials and the production of patient information resources that can be kept in clinics for access during consultations.

Despite the potential to improve the quality of healthcare, there is often inconsistent implementation of new research findings or practice recommendations [6]. Several barriers to implementation have been identified and various solutions offered [7, 8]. The process undertaken to create these standards of care is consistent with several of these recommendations. Importantly, there has been prominent patient and caregiver involvement throughout to ensure acceptability and appropriateness of the standards. We have also created plain language statements in collaboration with patient charity representatives. These will be disseminated by Myositis UK and via the active myositis social media community. From the perspective of the clinician we have facilitated dissemination and implementation of the standards of care by publishing this manuscript Open Access and have minimised the volume of information by presenting the standards as a single accessible table. Implementation measures, including an assessment of adoption and coverage of the standards of care, will be assessed after 12 months and reported through UKMYONET and Myositis UK.

We anticipate that these standards of care for adults with myositis will support clinicians, benefit patients and reduce variation by providing a benchmark of good practice that local services can be assessed against. As a consequence, we seek to mirror the improvements in practice seen in the management of stroke and epilepsy observed since the commencement of national audit programmes for these conditions [9, 10].

## Conclusion

Healthcare provision for patients with myositis is inconsistent. Consequently, many patients feel isolated and unsupported. Our service evaluation questionnaire identified common issues relating to diagnostic delays, poor access to specialists and allied healthcare professionals with experience in the care of patients with myositis, difficulties obtaining urgent medical advice, inadequate counselling regarding the risks of medications and poor management of extramuscular symptoms.

To address these issues, we have created patient-centred standards of care for adults with myositis. To facilitate implementation, plain language versions of the standards and an audit tool have been created. Implementation of these standards of care in the UK will promote consistent good practice and improve healthcare quality for patients with myositis.

## Additional files

Additional file 1: Section One – Copy of the Service Evaluation Questionnaire. Section Two – Draft standard of care statements and initial feedback. Section Three – Modified Delphi process. Section Four – Subsequent changes to statements. Section Five – Suggested Audit Standards. (DOCX 50 kb)

Additional file 2: Reviewer reports and AU response to reviewers. (DOCX 17 kb)

## Abbreviations

AGM: Annual General Meeting
ARMA: Arthritis and Musculoskeletal Alliance
DM: Dermatomyositis
GP: General practitioner
IIM: Idiopathic inflammatory myopathies
IQR: Interquartile range
PM: Polymyositis
SD: Standard deviation
UKMYONET: UK Myositis Network
