# Supplementary material for: Patient-centred standards of care for adults with myositis
Source: BMC Rheumatol. 2017 Nov 28;1:4. doi: 10.1186/s41927-017-0002-7 (PMC6383593; doi:10.1186/s41927-017-0002-7)
Supplement: Supplementary file 1 — Section One – Copy of the Service Evaluation Questionnaire. Section Two – Draft standard of care statements and initial feedback. Section Three – Modified Delphi process. Section Four – Subsequent changes to statements. Section Five – Suggested Audit Standards. (DOCX 50 kb) [file 41927_2017_2_MOESM1_ESM.docx]

**PATIENT-CENTRED STANDARDS OF CARE**

**FOR ADULTS WITH MYOSITIS**

***Supplementary Appendix***

Contents

[Section One – Copy of the Service Evaluation Questionnaire 2](#_Toc479328514)

[Section Two – Draft standard of care statements and initial feedback 9](#_Toc479328520)

[Section Three – Modified Delphi process 11](#_Toc479328521)

[Section Four – Subsequent changes to statements 13](#_Toc479328522)

[Section Five – Suggested Audit Standards 14](#_Toc479328523)

# Section One – Copy of the Service Evaluation Questionnaire

**Standards of Care: Idiopathic Inflammatory Myopathy**

Thank you for completing this service evaluation questionnaire. Please note, this is only intended for completion by members of Myositis UK who are currently being treated in the UK.

All data is collected anonymously and can in no way be traced back to you. Please answer openly and honestly.

We are very keen to improve the care delivered to patients with myositis and as such have developed this survey to understand your views on current service provision and how this could be improved. This is your opportunity to give feedback on issues that really matter to you about the care you receive.

**Background Details**

We need some background details to help us make sense of the feedback you receive. No identifiable data is collected.

1. What type of Idiopathic Inflammatory Myopathy (myositis) do you have?
   This should be your current diagnosis made by the specialist that looks after your myositis
   a
   □ Dermatomyositis (DM)
   □ Polymyositis (PM)
   □ Inclusion Body Myositis (IBM)
   □ Statin induced myopathy
   □ Necrotising myositis
   □ Other, please specify:______________________
2. What is your age? ___________*years*
   s
3. What is your sex?
   □ M □ F □ Prefer not to say
4. What is your ethnic group?
   □ White

□ Mixed / multiple ethnic groups
□ Asian / Asian British
□ Black / African / Caribbean / Black British
□ Prefer not to answer

1. What is the **first part** of your postcode?
    s
   ______________(e.g. WA13)

**Making a Diagnosis**

1. When was your myositis diagnosed?
   (please try and remember to the nearest year)
   s
   *____ /____ /________ (dd/mm/yyyy)*a
2. Regarding symptoms of myositis:
   What was the **first** symptom that you experienced? (select one)
   (if you had multiple symptoms beginning together, please choose the most prominent one)
   s
   *□ Muscle weakness
   □ Muscle aches or pain
   □ Skin rash (or any skin change)
   □ Fatigue or lethargy*

*□ Weight loss*

*□ Hair loss*

*□ Joint pains or swelling*

*□ Problems chewing or swallowing food*

*□ Shortness of breath*

*□ Chest pain or palpitations*

*□ Other, please specify:_______________*

1. With retrospect, how many months did you have symptom(s) related to myositis before seeking medical advice?
   a
   _____________*months*
2. Regarding symptoms that you have from myositis:

*a*a) What are the 3 most troublesome symptoms that you experience **NOW**? (select three)
a
b) What are the 3 most troublesome symptoms that you have **EVER** experienced? (select three)

*s*

***Now Ever*** *(tick 3 in each column)*
 *□ □ Muscle weakness*
 *□ □ Muscle aches or pain*
 *□ □ Skin rash (or any skin change)
 □ □ Fatigue or lethargy*
 *□ □ Weight loss*
 *□ □ Hair loss*
 *□ □ Joint pains or swelling*
 *□ □ Problems chewing or swallowing food*
 *□ □ Shortness of breath*
 *□ □ Chest pain or palpitations*
 *□ □ Other, please specify:
 _________________________*

1. How would you best describe the **pathway** taken to **diagnose** your myositis?

*a
□ GP (or other primary care practioner) visit with symptoms and subsequent referral to specialist.
□ Emergency admission to hospital (i.e. the GP was not your primary point of contact)
□ Direct appointment with a specialist (e.g. in private sector)
□ Other, please specify:
a*

*_________________________________________

_________________________________________*

a

1. If you initially visited the GP with symptoms, but the GP did not give you a diagnosis of myositis, how long did it take **until a referral** was sent to a specialist?
   *This is the length of time between first visiting your GP and being referred to a specialist. Please do not include the time spent waiting for an appointment after your GP had sent a referral.
   a
   □ I was referred immediately*

*□ less than 1 month*

*□ 1-3 months*

*□ 3-4 months*

*□ more than 4 months*

*□ N/A - GP was not involved in referral to specialist*

*□ N/A - GP made the diagnosis*

1. **After seeing the first specialist**, how long did it take (IN MONTHS) for you to be given (the correct) diagnosis?
   a
   ____________ *months
   □ N/A - GP made the diagnosis
   a*
2. **If** a referral to a specialist was made, which specialist was it?
   *(please only count the* ***first*** *specialist that you saw)*

*□ Rheumatologist*

*□ Neurologist*

*□ Orthopaedics*

*□ Extended scope physiotherapy*

*□ Ear, Nose & Throat (ENT)*

*□ Speech and Language Therapy (SALT)*

*□ Dermatologist*

*□ Chest/Respiratory*

*□ Renal
□ Other, please specify:______________________*

1. Was the **correct** diagnosis made by the **first** specialist that you saw?
   *□ Yes* *□ No*

*a*

1. **If no,** what other specialists did you then go on to see until a correct diagnosis was made (tick all that apply)?

*□ Rheumatologist*

*□ Neurologist*

*□ Orthopaedics*

*□ Extended scope physiotherapy*

*□ Ear, Nose & Throat (ENT)*

*□ Speech and Language Therapy (SALT)*

*□ Dermatologist
□ Chest/Respiratory
□ Renal
□ Other, please specify*:______________________
*□ N/A – correct diagnosis made by first specialist*

1. If you were initially given a different (incorrect) diagnosis by the GP or a specialist, what was it? (tick all that apply)
   *□ Motor Neuron Disease*

*□ Fibromyalgia*

*□ Chronic fatigue syndrome*

*□ "Old age"*

*□ Liver disease / hepatitis*

*□ Eczema or other skin condition*

*□ A different form of myositis (e.g. polymyositis instead of inclusion body myositis)
□ Arthritis
□ Other, please specify:______________________*

**How your current care is delivered**

We want to try and identify any gaps in provision for patients with myositis.

a

1. Regarding your myositis:
2. Which person made the **diagnosis**?
3. Who is the **main** person that **now** looks after your myositis?
   a

***Diagnosis Now*** *(tick one from each column)*

*□ □ GP
 □ □ Neurologist
 □ □ Rheumatologist
 □ □ Dermatologist
 □ □ A nurse specialist
 □ □ A GP with special interests (GPSE)
 □ □ No clear person
 □ □ Other:_______________________*

1. Are **other people also** involved in looking after your myositis? (tick all that apply)
   *(Do not include the main person that looks after your myositis)*

*□ Neurologist*

*□ Rheumatologist*

*□ GP*

*□ A GP with special interests (GPSE)*

*□ A nurse specialist or nurse consultant*

*□ Dermatologist*

*□ Renal (kidney doctor)*

*□ Respiratory (chest doctor)
□ Other(s), please specify:_______________
a*

1. How many times **in an average year** would you see the **main person** that looks after your myositis?
   *(enter 0 if you feel that there is no clear "main person" that looks after your myositis)
   a
   _________________ times
   a*
2. If you see a specialist for your myositis, do you feel that your usual GP and that person work well together to manage your condition?

*□ Yes □ No □ N/A
a*

1. If you see a specialist for your myositis, how far (in miles) do you have to travel to see that person?
   *(please only include distance on the way there - not the return journey)
   a
   ________________miles
   a*
2. Should the need arise, who would you contact if a problem with your myositis (or its treatment) required urgent review?

*□ Your GP*

*□ Your Specialist (e.g. via their secretary)*

*□ Your specialist directly (e.g. email or telephone)*

*□ Your Specialist Nurse (can be via a "helpline")*

*□ A&E department
□ Other, please specify:______________________
a*

1. How confident are you that you would be able to obtain urgent advice about your myositis (or its treatment) should the need arise?

*□ Very Confident*

*□ Confident*

*□ Neutral*

*□ Unconfident*

*□ Very Unconfident
a*

1. Do you have access to a local telephone advice line for issues relating to your myositis?

*□ Yes □ No □ Don't Know*

1. **If yes,** how satisfied are you with the service provided?

*□ Very Satisfied
□ Satisfied
□ Neutral
□ Dissatisfied
□ Very Dissatisfied
□ N/A*

*s*

1. How satisfied are you with access to the following services? **(inset number corresponding to your opinion from list below for each service)**

a
___ *Physiotherapy*___ *Occupational Therapy (OT)*___ *Speech and Language Therapy (SALT)*___ *Social Worker*___ *Specialist pharmacist*___ *Specialist nurse*___ *Psychologist / counsellor*___ *Rehab team*___ *Local support groups*___ *Orthotist*___ *Podiatrist*

*a*

**For each service, choose from:**

1. Very Satisfied
2. Satisfied
3. Neutral
4. Dissatisfied
5. Very Dissatisfied
6. No provision for this service locally
7. N/A

**Treatment**

1. What medication have you been given to treat of your myositis? (tick all that apply)

*□ Prednisolone (steroids)*

*□ Azathioprine □ Methotrexate*

*□ Mycophenolate □ Tacrolimus*

*□ Cyclosporine □ Cyclophosphamide*

*□ Rituximab □ Infliximab*

*□ Hydroxychloroquine*

*□ Plasma Exchange (PEx)*

*□ Intravenous Immunoglobulin (IVIG)*

*□ None
□ Other(s), please specify:____________________*

1. How satisfied are you that you have received adequate **counselling** about **side effects** and risks of your medication (as well as the proposed benefits)?

*□ Very Satisfied*
*□ Satisfied*

*□ Neutral*

*□ Dissatisfied*

*□ Very Dissatisfied
a*

1. What side effects (if any) have **you** experienced from medication used in the treatment of your myositis? (tick all that apply)
   *Please only include side effects from medication used in the treatment of your myositis (not any other conditions)*

*□ Weight gain* *□ Acne*

*□ Hair loss □ Anaemia
□ Fatigue □ Diarrhoea*

*□ Diabetes (includes sugar intolerance)*

*□ Infections (includes pneumonia and sepsis)*

*□ Fracture of bones (e.g. hip/spine)*

*□ Gastritis/stomach ulcer*

*□ Nausea/sickness/loss of appetite*
*□ Headache*

*□ Other(s), please specify:____________________*

*a*

1. At any point since being diagnosed with myositis have you been advised to receive flu and pneumonia **vaccination**?

*□ Yes*

*□ No*

*□ Cannot remember*

*a*

1. **If** you have been given prednisolone to treat your myositis, have you also been prescribed any of the following medication or had any of the following tests? (tick all that apply)
   a
   *□ "Bone Protection" (usually a bisphosphonate such as alendronate)*

*□ "Stomach Protection" (usually a "PPI" such as omeprazole)*

*□ Calcium/Vitamin D*

*□ Long term antibiotics*

*□ Had a DEXA (bone density) scan*

*□ None of the above*

1. How satisfied are you that the main person that looks after your myositis is up-to-date with the latest knowledge about myositis?

*□ Very Satisfied*

*□ Satisfied*

*□ Neutral*

*□ Dissatisfied*

*□ Very Dissatisfied*

*a*

1. How satisfied are you that the following areas **have been addressed** with regards to the treatment / management of your myositis?
   **(inset number corresponding to your opinion from list below for each service)**
   a
   *____ General health advice (e.g. regarding* *smoking, exercise, diet)*

*____ Support to "self manage" your condition*

*____ Psychological and emotional aspects*

*____ Pain control*

*____ Support regarding employment*

*____ Advice regarding fertility/pregnancy*

**For each aspect, choose from:**

1. Very Satisfied
2. Satisfied
3. Neutral
4. Dissatisfied
5. Very Dissatisfied
6. N/A
7. Have you been offered the opportunity to take part in a clinical trial for patients with myositis?

*□ Yes* *□ No* *□ Don’t know*

**Any other comments?**

__________________________________________________________________________________________________________

# Section Two – Draft standard of care statements and initial feedback

| Initial Draft Statements | Updated statements after input from Myositis UK | Updated statements after input from members of UKMYONET |
| --- | --- | --- |
| “GP’s should refer those with suggestive symptoms on to a specialist immediately, without trials of treatment or further investigation in the community” | “GP’s should refer those with suggestive symptoms of myositis on to a specialist after routine tests and in no longer than one week” | “GP's should identify patients presenting with features of myositis (e.g. muscle weakness, raised CK +/- rash) and refer to a specialist as soon as this diagnosis is considered” |
| “Myositis should be considered in those presenting to general practice with unexplained fatigue, joint or muscle pain or weakness” | **No change** | “Myositis should be considered in those presenting to general practice with unexplained fatigue, joint or muscle pain, or weakness. Testing of serum CK is a useful screening tool in this setting” |
| “A campaign to raise awareness of myositis amongst the public and professionals should take place” | **No change** | “A campaign to raise awareness of myositis amongst the public and healthcare professionals should take place to highlight "red flags" for myositis” |
| “Patients with myositis should be under the care of a specialist with specific expertise and experience in managing myositis” | **No change** | “Patients with myositis should be under the care of a specialist with specific expertise and experience in managing myositis, either directly or as part of a formal shared-care agreement with a local physician" |
| “Patients with myositis should remain under active specialist review (including being seen at least twice per year) for as long as their disease is active” | “Patients with myositis should remain under active specialist review. This should be responsive to need and for as long as their disease is active” | “Patients with myositis should continue to be periodically reviewed by a specialist for as long their disease is active or muscle strength continues to deteriorate" |
| “The services for patients with myositis should include integrated physiotherapy and occupational therapy” | “The services for patients with myositis should include integrated and ongoing specialist physiotherapy, occupational therapy and speech and language therapy” | “The services for patients with myositis should include access to ongoing specialist physiotherapy, occupational therapy and speech and language therapy. This could be integrated in to the specialist clinic or via a formal shared care agreement between specialist and non-specialist (local) therapists” |
| “Clear pathways should be defined for access to other services for patients with myositis, including Speech and Language Therapy, Orthotics, Podiatry, Psychology and Rehabilitation” | “There should be clearly defined pathways for facilitating access to local services. This should include liaison between specialist and community therapists (and might include provision of information sheets or DVD’s containing specialist advice)” | “There should be formal agreement defining the roles, responsibilities and interaction of 'specialist' and 'local' healthcare professionals. This might include provision of a personalised hand-held patient record to support communication between all concerned parties" |
| “Those caring for patients with myositis should signpost relevant patient information resources and charity groups” | **No change** | “Patients with myositis should be provided with information resources and patient group contact details. This could form part of a patient hand-held record” |
| “When considering starting patients with myositis on immunosuppression, detailed discussion regarding the potential benefits and possible harms must take place. This might include access to a specialist pharmacist” | **No change** | “When considering starting patients with myositis on immunosuppression, detailed discussion regarding the potential benefits and possible side effects must take place. This could be reinforced by other members of the multidisciplinary team (e.g. pharmacist). In addition, formal shared-care agreements with GPs should also be in place” |
| “Patients with myositis should have access to a myositis specialist nurse service” | **Majority voted to remove this statement** | **Negative feedback from majority of respondents** |
| “Clear local protocols should be defined that outline how patients with myositis should seek urgent advice should the need arise. This might include provision of a dedicated telephone advice line.” | **No change** | “There should be clear protocols defining how patients with myositis should seek urgent advice. For example, the specialist centre might provide a dedicated telephone advice line for patients and other healthcare professionals." |
| “Services for patients with myositis should provide holistic care that addresses physical and psychological aspects of disease and its social implications (e.g. difficulties with employment)” | **No change** | **No change** |
| “Services for patients with myositis should take special account of issues affecting women of childbearing age (e.g. fertility advice)” | “Services for patients with myositis should specifically take into account fertility issues that might affect patients” | “Services for patients with myositis should specifically consider issues with fertility and pregnancy that might affect patients” |
| “Patients with myositis commonly experience pain and fatigue. Specialist should specifically enquire about the presence of these symptoms and actively manage them. This might include local pathways of guidelines and involvement of other services (e.g. the chronic pain team)” | **No change** | “Patients with myositis commonly experience pain and fatigue. Specialists and the MDT should specifically enquire about the presence of these symptoms and actively manage them" |
| “Patients with myositis should be offered participation in clinical trials as part of routine practice” | **No change** | **No change** |

# Section Three – Modified Delphi process

| **Considered Statements** | **Mean Agreement Level -  Delphi Round 1**  ***Updated statement where appropriate*** | **Mean Agreement Level -**  **Delphi Round 2** |
| --- | --- | --- |
| “GP's should identify patients presenting with features of myositis (e.g. muscle weakness, raised CK +/- rash) and refer to a specialist as soon as this diagnosis is considered” | 8.76 | - |
| “Myositis should be considered in those presenting to general practice with unexplained fatigue, joint or muscle pain, or weakness. Testing of serum CK is a useful screening tool in this setting” | 8.16  “Myositis should be considered in patients with unexplained weakness, fatigue, rash, myalgia or arthralgia. Testing of serum CK is a useful screening tool, but can be normal in some scenarios” | 9.13 |
| “Patients with myositis should be under the care of a specialist with specific expertise and experience in managing myositis, either directly or as part of a formal shared-care agreement with a local physician” | 9.52 | - |
| “Patients with myositis should continue to be periodically reviewed by a specialist for as long their disease is active or muscle strength continues to deteriorate" | 9.08 | - |
| “The services for patients with myositis should include access to ongoing specialist physiotherapy, occupational therapy and speech and language therapy. This could be integrated in to the specialist clinic or via a formal shared care agreement between specialist and non-specialist (local) therapists” | 9.20 | - |
| “Patients with myositis should be provided with information resources and patient group contact details. This could form part of a patient hand-held record” | 8.48  “Patients with myositis should be signposted to appropriate information resources and patient groups” | 9.32 |
| “When considering starting patients with myositis on immunosuppression, detailed discussion regarding the potential benefits and possible side effects must take place. This could be reinforced by other members of the multidisciplinary team (e.g. pharmacist). In addition, formal shared-care agreements with GPs should also be in place” | 8.80 | - |
| “There should be clear protocols defining how patients with myositis should seek urgent advice. For example, the specialist centre might provide a dedicated telephone advice line for patients and other healthcare professionals." | 8.92 | - |
| “Services for patients with myositis should provide holistic care that addresses physical and psychological aspects of disease and its social implications (e.g. difficulties with employment)” | 8.68 | - |
| “Patients with myositis commonly experience pain and fatigue. Specialists and the MDT should specifically enquire about the presence of these symptoms and actively manage them" | 8.20  “Healthcare professionals should specifically address extra-muscular symptoms such as pain, fatigue and depression at each consultation”* | 8.73 |
| “Patients with myositis should be offered participation in clinical trials as part of routine practice” | 8.84 | - |
| ***Statements that did not reach consensus after second round*** | | |
| “A campaign to raise awareness of myositis amongst the public and healthcare professionals should take place to highlight "red flags" for myositis” | 7.64  “A clinical ‘red-flag’ approach should be used to prompt timely consideration of the diagnosis of myositis” | 6.91 |
| “There should be formal agreement defining the roles, responsibilities and interaction of 'specialist' and 'local' healthcare professionals. This might include provision of a personalised hand-held patient record to support communication between all concerned parties" | 7.24  “Patients with myositis should be provided with a patient-centred care plan to facilitate communication between multidisciplinary team members” | 8.23 |
| “Services for patients with myositis should specifically consider issues with fertility and pregnancy that might affect patients” | 7.70  “Fertility and pregnancy services should be readily accessible to myositis patients who are attempting to conceive or who are already pregnant” | 8.10 |

* The word “depression” was added to this statement between round 1 and round 2 of the Delphi exercise. In summary, patients completing the Standards of Care survey highlighted that the psychological aspects of disease were poorly addressed (only 23.7% felt satisfied). After analysing the results from round 1 of the Delphi exercise, it was felt important by the steering team to include reference to depression in an attempt to address this.

# Section Four – Subsequent changes to statements

Minor changes to some of the standard of care statements were made after the consensus exercise. The purpose of this was to improve their readability of each statement and to emphasise the key message. Changes were agreed amongst the steering team. In some instances, the statement has been separated in to a principle statement (usually the first sentence) and sub-statements (usually the following statements, which mainly contained clarifications and more general explanations). Italicisation of some parts of each statement has also been added to emphasise the key messages and improve readability. A log of the changes made (except italicisation) according to final statement number is as follows:

1. Statement 1: The second sentence has been made a sub-statement.
2. Statement 3: The second sentence has been made a sub-statement and words “this could be…” added to improve readability.
3. Statement 4: In the first sentence, “reviewed by a specialist” has been changed to “reviewed by a myositis specialist” (as defined in the preceding statement: “…with specific expertise and experience in managing myositis”). The same sub-statement from Statement 3 has also been added (“…This could be either directly or as part of a formal shared-care agreement with a local physician”) to improve clarity.
4. Statement 5: The second sentence has been made a sub-statement.
5. Statement 6: The second sentence has been made a sub-statement.
6. Statement 7: The second and third sentences have been made sub-statements. The words “In addition” have been removed to improve readability.
7. Statement 9: The text within parenthesis has been updated (from “e.g. difficulties with employment”, to “For example, this may include difficulties with employment”) and made a sub-statement.

# Section Five – Suggested Audit Standards

| **Statement Number** | **Patient-Centred Standards of Care** | ***Suggested* Audit Standard** |
| --- | --- | --- |
| 1 | Myositis should be considered in patients with *unexplained weakness, fatigue, rash, myalgia or arthralgia*   - Testing of serum *CK is a useful screening tool*, but can be normal in some scenarios | n/a |
| 2 | GPs should identify patients presenting with features of myositis (e.g. muscle weakness, raised CK +/- rash) and refer to a specialist *as soon as this diagnosis is considered* | n/a |
| 3 | Patients with myositis should be *under the care of a specialist with specific expertise and experience in managing myositis*   - This could be either directly or as part of a formal shared-care agreement with a local physician | n/a |
| 4 | Patients with myositis should continue to be *periodically reviewed by a myositis specialist* for as long their disease is active or muscle strength continues to deteriorate   - This could be either directly or as part of a formal shared-care agreement with a local physician | At least 90% of my patients with active myositis have a review appointment within the next 6 months |
| 5 | The services for patients with myositis should include access to *ongoing specialist* physiotherapy, occupational therapy and speech and language therapy   - This could be integrated in to the specialist clinic or via a formal shared-care agreement between specialist and non-specialist (local) therapists | At least 90% of my patients have access to a *specialist* physiotherapist, occupational therapist and speech and language therapist (where required) |
| 6 | There should be *clear protocols defining how* patients with myositis should *seek urgent advice.*   - For example, the specialist centre might provide a *dedicated telephone advice line* for patients and other healthcare professionals | At least 90% of my patients with myositis are aware of a clearly defined pathway for seeking urgent advice (should the need arise) |
| 7 | When considering starting patients with myositis on immunosuppression*, detailed discussion regarding the potential benefits and possible side effects must take place*.   - This could be reinforced by other members of the multidisciplinary team (e.g. pharmacist) - Formal shared-care agreements with GPs should also be in place | In at least 90% of my patients with myositis the benefits and risks of proposed treatments were discussed and documented |
| 8 | Healthcare professionals should specifically address *extra-muscular symptoms* such as pain, fatigue and depression at each consultation | At their last review, at least 90% of my patients with myositis were asked about pain, fatigue and depression |
| 9 | Services for patients with myositis should provide *holistic care* that addresses *physical and psychological aspects* of disease and its *social implications*   - For example, this may include difficulties with employment | At least 90% of my patients with myositis have access to support with psychological and non-medical aspects of their disease (where required) |
| 10 | Patients with myositis should be *signposted to appropriate information resources and patient groups* | At least 90% of my patients with myositis have been signposted to appropriate information resources and patient groups |
| 11 | Patients with myositis should be *offered participation in clinical trials* as part of routine practice | At least 90% of my patients with myositis have been offered access to clinical trials (where appropriate). |
